# Imaging-Based Deep Graph Neural Networks for Survival Analysis in Early Stage Lung Cancer Using CT: A Multicenter Study

**DOI:** 10.3389/fonc.2022.868186

**Published:** 2022-07-13

**Authors:** Jie Lian, Yonghao Long, Fan Huang, Kei Shing Ng, Faith M. Y. Lee, David C. L. Lam, Benjamin X. L. Fang, Qi Dou, Varut Vardhanabhuti

**Affiliations:** ^1^ Department of Diagnostic Radiology, Li Ka Shing Faculty of Medicine, The University of Hong Kong, Hong Kong, Hong Kong SAR, China; ^2^ Department of Computer Science, The Chinese University of Hong Kong, Hong Kong, Hong Kong SAR, China; ^3^ Faculty of Medicine, University College London, London, United Kingdom; ^4^ Department of Medicine, Li Ka Shing Faculty of Medicine, The University of Hong Kong, Hong Kong, Hong Kong SAR, China; ^5^ Department of Radiology, Queen Mary Hospital, Hong Kong, Hong Kong SAR, China

**Keywords:** lung cancer, graph convolutional networks, cox proportional-hazards, survival prediction, lung graph model

## Abstract

**Background:**

Lung cancer is the leading cause of cancer-related mortality, and accurate prediction of patient survival can aid treatment planning and potentially improve outcomes. In this study, we proposed an automated system capable of lung segmentation and survival prediction using graph convolution neural network (GCN) with CT data in non-small cell lung cancer (NSCLC) patients.

**Methods:**

In this retrospective study, we segmented 10 parts of the lung CT images and built individual lung graphs as inputs to train a GCN model to predict 5-year overall survival. A Cox proportional-hazard model, a set of machine learning (ML) models, a convolutional neural network based on tumor (Tumor-CNN), and the current TNM staging system were used as comparison.

**Findings:**

A total of 1,705 patients (main cohort) and 125 patients (external validation cohort) with lung cancer (stages I and II) were included. The GCN model was significantly predictive of 5-year overall survival with an AUC of 0.732 (p < 0.0001). The model stratified patients into low- and high-risk groups, which were associated with overall survival (HR = 5.41; 95% CI:, 2.32–10.14; p < 0.0001). On external validation dataset, our GCN model achieved the AUC score of 0.678 (95% CI: 0.564–0.792; p < 0.0001).

**Interpretation:**

The proposed GCN model outperformed all ML, Tumor-CNN, and TNM staging models. This study demonstrated the value of utilizing medical imaging graph structure data, resulting in a robust and effective model for the prediction of survival in early-stage lung cancer.

## Introduction

Lung cancer is the leading cause of cancer-related mortality around the world, accounting for more than 1.80 million deaths in 2020 (1). It is commonly accepted that early detection and treatment improve patients’ outcomes (2). Although medical imaging technologies such as computed tomography (CT) scan have made significant advances in recent years, accurate diagnosis, particularly of early lung cancer on CT images, and corresponding individual survival prediction remains a challenge. In recent years, using machine learning and deep learning approaches have recently become a promising tool for helping radiologists and physicians improve detection and prognostication (3, 4).

For example, Jin et al. (5) used the convolution neural network (CNN) as a classifier in their computer-aided diagnosis method to detect lung pulmonary nodules on CT images, achieving an accuracy of 84.6% and sensitivity of 82.5% on the Lung Image Database Consortium image collection (LIDC-IDRI). Sangamithraa et al. (6) applied a K-mean learning algorithm for clustering-based segmentation and a back propagation network for classification to achieve an accuracy of 90.7% on their own dataset. Besides, She et al. (7) applied deep learning models with radiomic features as input and achieved a C-index of 0.7 for survival prediction after surgery. While the approaches described above achieved a good level of prediction performance for nodule detection and prognosis, their models have the following limitations. First, the majority of studies used small patient numbers, which resulted in the respective models only performing well on specific datasets, thus limiting generalizability. Second, most of the previous research used strict criteria for their input images; for example, some pre-trained models performed well only on contrast-enhanced CT, although there was a considerable amount of non-contrast CT being used in practice. Additionally, a substantial number of current machine learning models with radiomic features required expert radiologists to manually segment tumors (8–11), which is time consuming, and the relevant findings heavily relied on radiologists’ experience. Moreover, the majority of the models was constructed using pixels that focused exclusively on the tumor, without reference to surrounding structures or patient-specific clinical data, despite the fact that they may also contain disease-related information. In clinical practice, clinicians use that additional information to make treatment decisions and risk stratify patients for more accurate treatment and prognosis (12). In essence, these additional features are analogous to “domain knowledge,” which has been underutilized in prior research.

Graph convolutional neural network (GCN) (13) is an emerging technique used to tackle data with graph structures, owing to its effectiveness to model relationships across different factors. In graph, nodes are regarded as different entities, while edges present the relationship between each pair of nodes. This approach is unique in that it is able to elegantly incorporate connections from various features. In recent years, graph presentation has been widely used, for instance, social network analysis, language translation, and point cloud, also in the medical field such as vascular segmentation (14) and airway segmentation (15) due to the fact that some organs and systems within the human body are inherently based on graph or network structures (e.g., vascular structures such as retinal vessels) (16, 17). Lungs also inherently have graph structures (18) if we regard every lung lobe as nodes connected by the airway which can be regarded as edges. In theory, the relationship between different parts of the lungs can be modeled and GCN can be applied on lung CT images to tackle clinical problems.

In this study, we developed a graph representation to summarize information of stage I and II lung cancer patients and to forecast their 5-year overall survival rates using CT and clinical data. This study demonstrated the utility of applying medical domain knowledge to create graph structure data and making predictions with state-of-the-art graph convolutional neural network models, which provided a robust and effective model for early stage lung cancer survival prediction.

## Materials and methods

### Data Description

The Institutional Review Board of Shanghai Pulmonary Hospital has approved this retrospective study protocol and waived the requirement for informed consent for all included patients. The main cohort of the study included consecutive patients who underwent surgery for early stage non-small cell lung cancer (NSCLC) from January 2011 to December 2013. The inclusion criteria were as follows: (I) pathologically confirmed stage (I) and (II) NSCLC, (II) availability of preoperative thin-section CT image data, and (III) complete follow-up of survival data. Patients receiving neoadjuvant therapy were excluded. An external validation set of 125 patients who met our criteria were also retrieved from the NSCLC Radiogenomics (19) dataset (please refer to original reference for related data information). We only used the one single CT image when patient was diagnosed as NSCLC. Both contract and non-contrast CT were included.

### Scanning Parameters

The CT scans were performed using Somatom Definition AS+ (Siemens Medical Systems, Germany) and iCT256 (Philips Medical Systems, Netherlands). Detailed scanning parameters can be found in Supplementary Material I. Intravenous contrast was given according to institutional clinical practice. Relevant clinical data were manually extracted from medical records. The follow-up data were acquired from outpatient records and telephone interviews. Overall survival (OS) was defined as the time interval between the date of surgery and the date of mortality or the last follow-up. Recurrence-free survival (RFS) was measured from the time of surgery to the date of recurrence or death or last follow-up (more details can be found at Supplementary Material II).

### Lung CT images Segmentation

**Figure 1:**
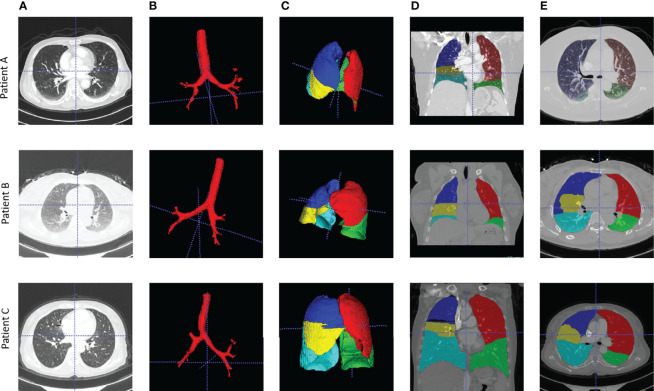
Examples of airway and lung lobes segmentation. **(A)** Patient raw CT scan; **(B)** Airway segments produced by region-growing algorithm; **(C)** lung lobes segments, 3D; **(D)** Lung lobes segments, x-axial 2D; **(E)** Lung lobes segments, z-axial 2D.

Lung CT segmentation is a necessary first step in analyzing the pulmonary structures, and it has been regarded as a necessary prerequisite for accurate CT image analysis tasks (20). Before segmentation, every CT data were preprocessed with slice thickness of 1 mm and matrix of 512×512 mm, following normalization. Several image segmentation approaches were adopted in this project to ensure accurate preparation for the graph modeling and analysis. The 3D airways were segmented using an adaptation of the region-growing method (21), where we randomly picked a seed point from non-background region in the CT image, and neighbor pixels were examined until the borders. The generated airway segments was then skeletonized with a skeleton algorithm (22) to obtain the main structure of the airways. We then applied a searching algorithm to find the four most important points, namely, the root point, the center point, the left point, and the right point (see Supplementary Material III), and segmented a bounding box of 64×64×64 from the original CT to represent the main properties of the corresponding area of the tissue around the airway. Furthermore, for each patient, a public pre-trained UNet (23) model called lung mask (24) was adapted to segment the five lung lobes. In the last step, tumor image was cropped with the bounding box from CT by using the corresponding annotation information provided by radiologists. For each patient, this resulted in images for 10 separate lung structures, namely, five lung lobes, four airway landmarks, and one tumor segment (**Figure 1**).

### Graph Building and Graph Convolutional Neural Network Architecture

The very first step in this study is to build meaningful structure of the lung graphs, particularly defining the vertices and their connections. To use the natural structure of the lung, we considered the four airway landmarks and five lung lobe segments as nodes in each graph, and all nodes were connected in their natural ways. To emphasize the significance of the tumor, we added a tumor node to each patient’s lung graph, and the tumor node was connected to their corresponding lobes in which the tumor was located. For example, if the tumor was detected on the left upper lobe, the tumor node will be connected to the left upper lobe node. Each CT were modeled as a 10-node graph for further analysis.

**Figure 2:**
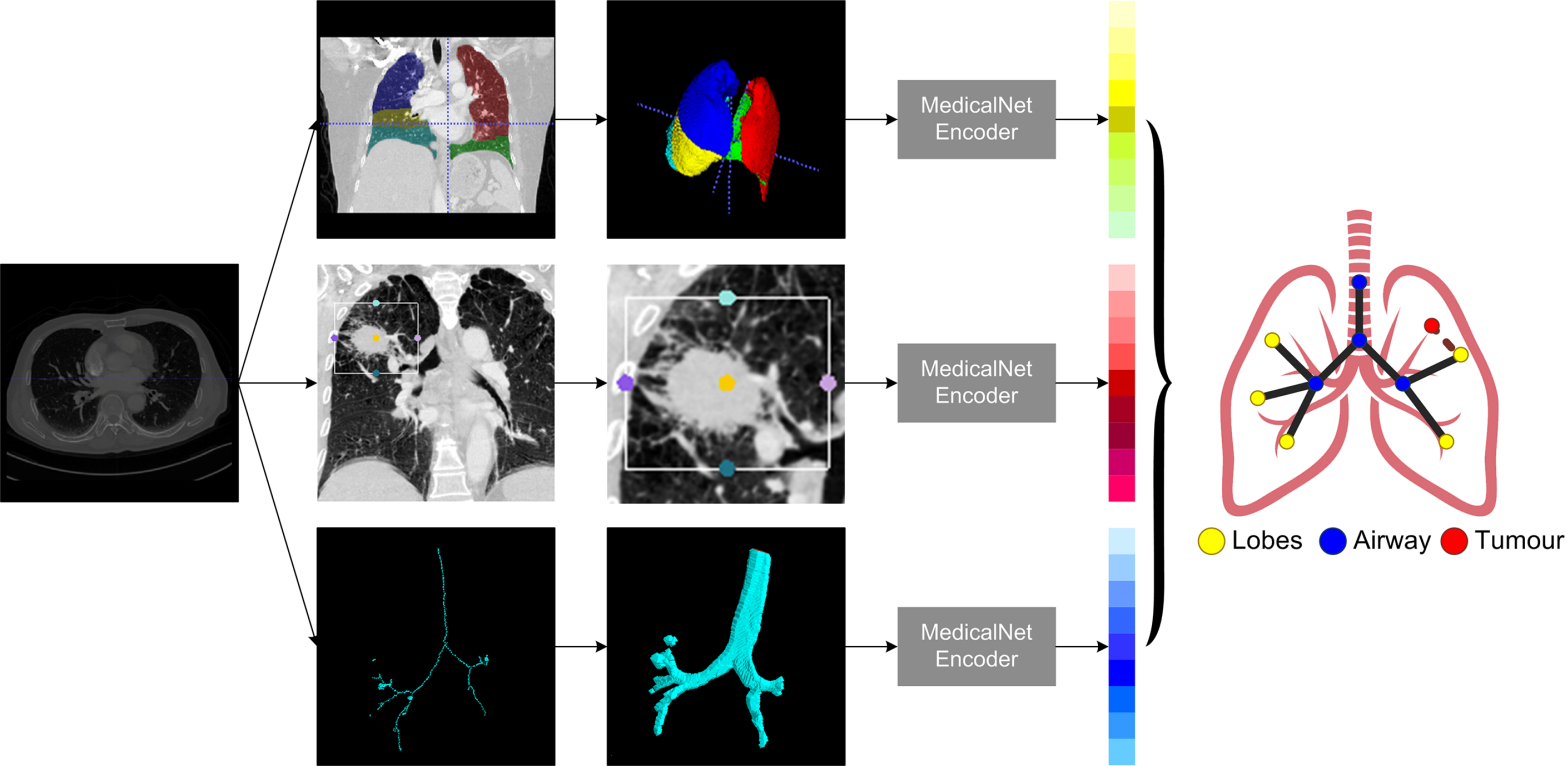
The pipeline of building patients’ lung graph building.

For each patient node, a feature vector should be defined to represent the corresponding properties. In this study, we used the pre-trained MedicalNet (25) to get the relevant image features, followed by an average pooling layer to reduce the dimension space to one dimension (1D). The MedicalNet is a collection of ResNet (26) models that have been pre-trained on a variety of large medical datasets and have demonstrated exceptional performance on medical deep learning tasks such as organ segmentation and nodule detection. To keep the feature vectors simpler and more representative, a linear ridge transform method was used to lower the dimension of each node’s feature vector from 1,024 to 96 as the final feature vectors on patients’ lung graphs (**Figure 2**).

The goal of GCN is to learn the graph or node embedding using the node’s neighborhood information with a neural network. Recently, an inductive framework called GraphSage (27), which allows updating node features by sampling and aggregating information from the neighboring nodes, achieved promising performance among various graph neural network topologies on networks. This network was deemed highly suitable for our study, as our lung CT graph was designed to emphasize the interaction within different parts of a patient’s lung structure. Therefore, we designed a survival prediction graph neural network predictor composed of SageConv blocks, a mean-readout layer, and a fully connected layer. This model will output a survival label for each patient graph. In detail, the SageConv block consists of a GraphSage Convolution layer with a long short-term memory (LSTM) aggregator, a ReLu activation layer, a dropout layer, and a layer normalization function, which are all efficiently extracting the diagnosis knowledge from the patient graph. The entire model was trained on two GPU nodes in parallel, with a total training epoch of 100. We set a reduced learning rate method to find the optimal training with an initialization value of 0.01 and a minimal value of 0.00001 in order to train the model effectively. In addition, to avoid overfitting when training the model, a weight decay function with value of 0.00005 was added. In order to get the best-performed graph structure, we tested the number of layers of SageConv blocks from 1 to 4, and only the best-performed model was reported.

### Experiment Design and Statistical Analysis

To demonstrate the performance of the GCN model on lung cancer survival prediction, a set of experiments were implemented on our dataset. The whole patient cohort was randomly split as training, validation, and testing sets with a ratio of 75% (1278), 12.5% (213), and 12.5% (214) stratified for survival, keeping the survival rate almost equal when splitting the dataset, and there was no significant difference in age and sex among each subset (**Table 1**). We evaluated the performance of the lung graph model by using the area under the receiver operating characteristics (AUC) score, sensitivity, specificity, and precision scores. In order to put emphasis on the model and not to miss the true positive cases, we also added F_2_ score (28) as one of the metrics. All relevant results can be found in **Supplementary Table S1**. Wilcoxon rank sums tests were performed to compare performance with baseline model.

In order to see the performance of this graph presentation method with both current clinical assessment and novel deep learning methods, we selected the standard clinical model (TNM staging), commonly used clinical Cox proportional-hazard model, traditional machine learning methods, along with a state-of-the-art deep learning model to make comparison:

1) TNM staging model: using T, N, and M information to make prediction (baseline model I);

2) a Cox proportional-hazard model: using the clinical features (patient sex, age, tumors size, tumors staging, and histology information) as input (baseline model II);

3) a set of machine learning (ML) models: using 103 tumors radiomic features as input (baseline model III), with only the best performer used as the baseline model to be compared;

(4) Tumor-CNN: using individual’s tumor segments as input for a ResNet-50 deep neural network.

All models were trained and tested on the same dataset to predict an individual patient’s 5-year overall survival, and the best results were reported in comparison to GCN model. We further implemented the survival analysis with Kaplan–Meier estimates for low- and high-risk patients based on the scores predicted by the best three performing models on the testing set, along with a log-rank test. Hazard ratio of our GCN biomarker was calculated by a Cox proportional-hazard model. Finally, a subanalysis was implemented to evaluate the GCN model’s performance for predicting overall survival and relapse-free survival on stage I and II patients dataset separately.

All experiments were performed using Python 3.7. The statistics analysis was implemented with the package of Pandas (version 1.3.0) and statistics (version 3.4). Radiomic features were calculated with the PyRadimics package (version 3.0.1). The machine learning models were implemented with the library of Scikit-Learn (version 0.24). Both the Cox regression and the Kaplan–Meier curve were calculated by using the Lifelines package (version 0.26.03). The whole GCN structure was implemented using Deep Graph Library (version 0.6.1) and PyTorch (version 1.8.0).

## Results

### Patient Information Statistics

A total of 1,705 NSCLC patients were included in the main cohort. There were 1,010 men (59.2%) and 695 women (40.8%) with a median age of 61 years (range: 55–66 years). The median follow-up time is 70.9 months. Of these, 145 patients (8.5%) received sub-total lobectomy, 1,472 patients (86.3%) underwent lobectomy, 66 patients (3.9%) received bi-lobectomy, 21 patients (1.2%) underwent pneumonectomy, and one patient received sub-total lobectomy of one lobe plus total lobectomy of another lobe. Tumors were most commonly located in the upper lobe [419 left upper lobe (LUL), 24.6%, and 565 right upper lobe (RUL), 33.1%]. A total of 1,235 tumors (72.4%) were diagnosed as adenocarcinoma, and 391 tumors (22.9%) were squamous cell carcinoma. The distribution of pathological stages was as follows: stage IA in 791 patients (46.4%), stage IB in 607 patients (35.6%), stage IIA in 133 patients (7.8%), and stage IIB in 174 patients (10.2%). In the whole main cohort, the 3-year OS and RFS were 98.4% and 81.1%, respectively, and the 5-year OS and RFS were 78.2% and 74.2%, respectively.

There were 33 (26.4%) female and 92 (73.6%) male patients in the external validation dataset, with a median age of 69 (range, 43–87 years). Tumors in the upper lobe were also the most common [41 at right upper lobe (RUL), 32.8%, and 32 at left upper lobe (LUL), 25.6%]. There were 97 patients with adenocarcinoma and 26 with squamous cell carcinoma among them. The pathological stages were distributed as follows: stage IA in 40 patients (32.0%), stage IB in 23 patients (18.4%), stage IIA in 45 patients (36.0%), and stage IIB in 17 patients (13.6%). The RFS was 74.4%, while the 5-year OS was 63.2%. **Table 1** provides the rest of the patient’s detailed information.

**Table 1:** Feature distribution in the total patient cohorts, training and validation cohorts, and the test cohorts.

		Patients Characteristics(*n* = 1,705)	TRAIN and VAL(n = 1,492)	Test (n = 213)	EXTERNAL (n= 125)
Feature	Content	Mean, SD, 95% CI/Count and percentage (%)			
**Age**	Age	60.6, 8.8, (CI: 60.2- 61.0)	60.6, 8.7, (CI: 60.1- 61.0)	60.7, 9.5, (CI: 59.4- 62.0)	69.0, 8.90, (CI: 67.4- 70.5)
**Sex**	Female No. (%); Male No. (%)	695 (33.3); 1010 (66.7)	602 (33.3); 890 (66.7)	93 (33.3); 120 (66.7)	33 (26.4); 92 (73.6)
**Resection**	Sublobar Resection No. (%); Lobectomy No. (%); Bilobectomy No. (%); Pneumonectomy No. (%)	146 (8.6); 1472 (86.3); 66 (3.9); 21 (1.2)	123 (8.2); 1,292 (86.6); 59 (3.95); 18 (1.2)	23 (10.8); 180 (84.5); 7 (3.3); 3 (1.4)	/
**Histology**	Adenocarcinoma No. (%); Squamous Cell Carcinoma No. (%); Others No. (%)	1,235 (72.4); 391 (22.9); 79 (4.6)	1,072 (71.4); 351 (23.5); 69 (4.6)	163 (76.5); 40 (18.8); 10 (4.7)	97 (77.6); 26 (20.8); 2 (1.6)
**Tumor Size**	Tumor Size	2.66, 1.37, (CI: 2.60- 2.73)	2.68, 1.38, (CI: 2.61- 2.75)	2.55, 1.25, (CI: 2.38-2.71)	/
**pTNM stage**	Stage I No. (%); Stage II No. (%)	1,398 (82.0); 306 (18.0)	1,219 (81.7); 273 (18.3)	179 (84.0); 34 (16.0)	63 (50.4); 62 (49.6)
**RFS Status**	RFS No. (survival %)	1,243 (72.9)	1,089 (73.0)	154 (72.3)	93 (74.4)
**RFS Month**	RFS Month	57.6, 24,4, (CI: 56.4- 58.7)	57.5, 24.5, (CI: 56.2- 58.7)	58.4, 23.4, (CI: 55.2- 61.5)	/
**OS Status**	OS No. (survival %)	1,333 (78.2)	1,166 (78.2)	167 (78.4)	79 (63.2)
**OS Month**	OS Month	62.5, 19.8, (CI: 61.6- 63.5)	62.4, 19.9, (CI: 61.4- 63.4)	63.4, 18.4, (CI: 60.9- 65.9)	/

### Model Evaluation

As shown in **Table 2**, the Cox modeling and ML radiomic feature baseline models showed poor performance on the testing set. The best performing ML radiomic model was from the decision tree (DT) model, while other ML models such as SVM, linear classification, K-means, LASSO, and KNN methods had worse performance than the DT predictor. The Tumor-CNN model had a significantly improved performance (AUC=0.614; 95% CI: 0.519–0.710; p < 0.05) compared with the two baseline models, although the TNM method performed better (AUC=0.633; 95% CI: 0.539–0.728; p < 0.005). The GCN model achieved the highest AUC score of 0.732 (95% CI: 0.643–0.821; p < 0.0001) among all models in survival prediction for early-stage lung cancer. On external validation dataset, our GCN model achieved the AUC score of 0.678 (95% CI: 0.564–0.792; p < 0.0001).

**Table 2:** Performance for each model based on AUC scores and the Wilcoxon rank-sum tests.

ML models	AUC scores (95% CI)	p-values
**CPH Model**	0.549 (0.454–0.645)	.45
**DT-radiomics**	0.572 (0.476–0.668)	.33
**Tumor-CNN**	0.614 (0.519–0.710)	.02
**TNM**	0.633 (0.539–0.728)	.002
**GCN**	0.732 (0.643–0.821)	< 0.0001

**Figure 3:**
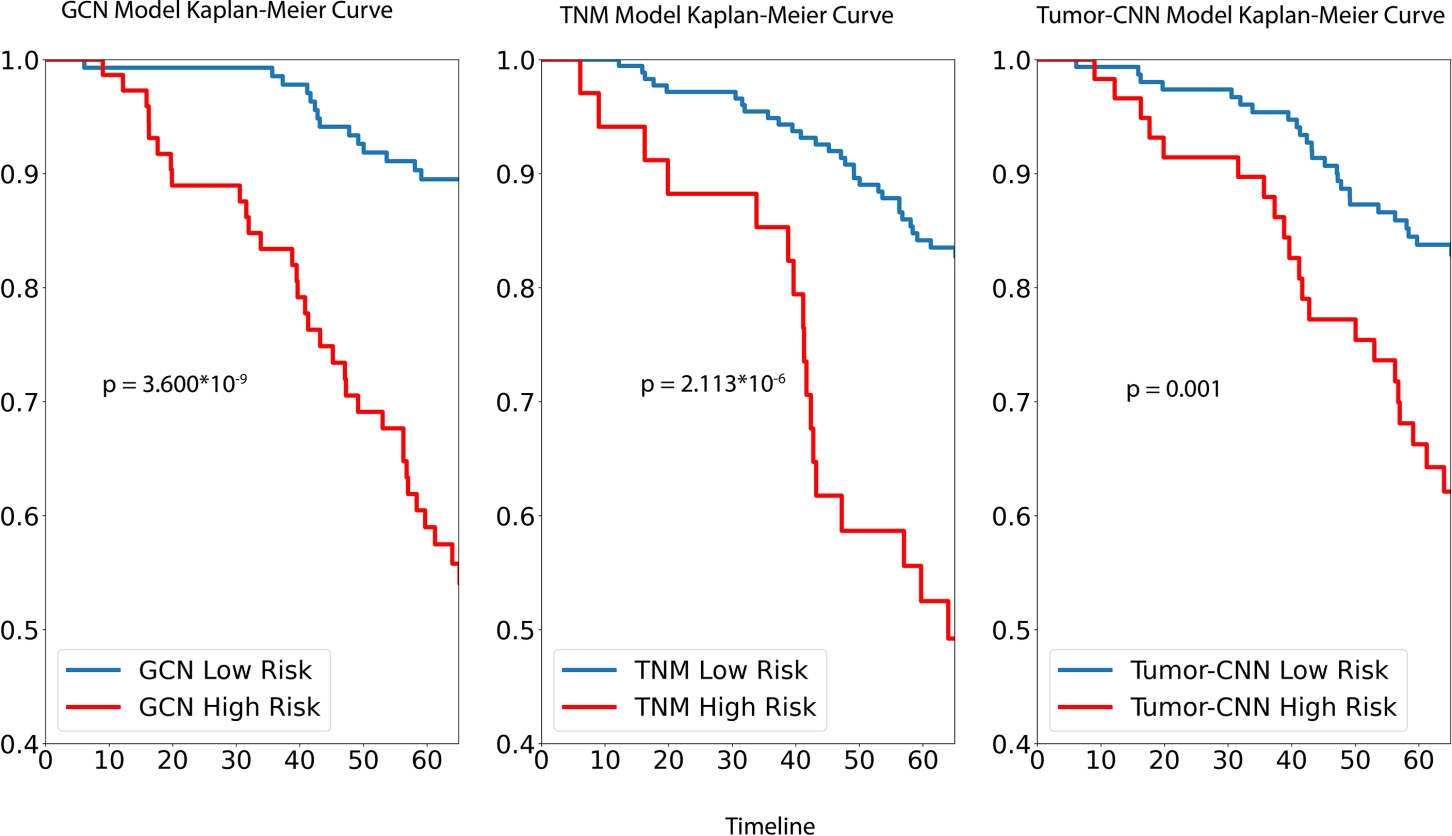
Performance of GCN, TNM and Tumor-CNN models on testing dataset.

For survival analysis, both GCN the cancer staging system and Tumor-CNN shared a similar trend and, based on Kaplan–Meir analysis, were able to demonstrate significant separation of high- and low-risk groups (**Figure 3**), while the p-value of the log rank sums test suggested that GCN has a stronger separation ability compared with the others. Comparable results were found in the prediction of 5-year survival outcomes with the hazard ratios, respectively, for GCN (HR = 5.41; 95% CI: 2.32–10.14; p=0.000014), and TNM (HR = 3.85; 95% CI: 1.91–7.02; p=0.00015).

**Figure 4:**
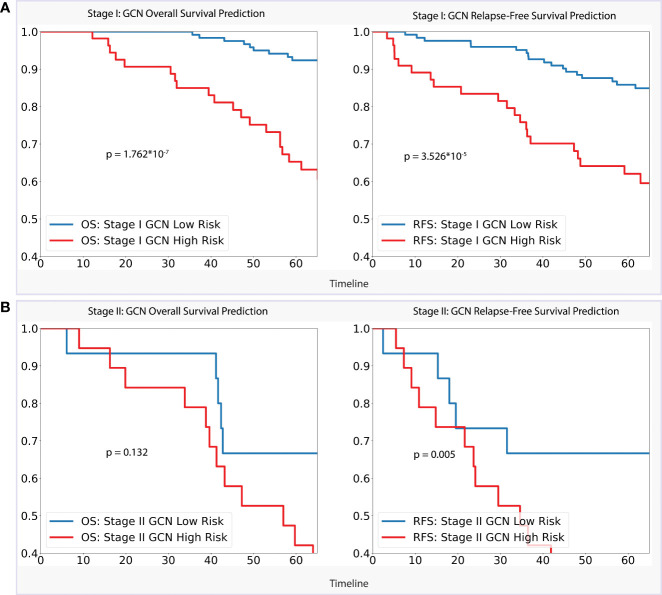
**(A)**. Stage I Analysis: Performances of GCN models on OS prediction and RFS prediction separately; **(B)**. Stage II Analysis: Performances of GCN models on OS prediction and RFS prediction separately.

For the stage I dataset (n=179) analysis, as per **Figure 4**, our GCN model achieved a clear separation of low- and high-risk groups in 5-year overall survival prediction (p < 0.0001) and relapse-free survival prediction (p < 0.0001), with AUC of 0.728 (CI: 0.618–0.839) and 0.660 (CI: 0.555–0.757) separately. Referencing stage II (n=55), the model showed slightly weaker performance of separation for 5-year overall survival (AUC = 0.647, CI: 0.461–0.834, p = 0.132) comparing with stage I dataset, while better performance for relapse-free survival prediction (AUC = 0.702, CI: 0.532–0.877, p < 0.01) was achieved.

## Discussion

Prediction of survival of early-stage lung cancer patients remains a challenging task. In this paper, we proposed a graph-based method to represent a patient’s lung CT images and applied the state-of-the-art graph convolutional neural network to improve 5-year survival predictions for individual patients. In previous studies, especially for some small size cohorts, the radiomic feature methods (our baseline models) were commonly used. The results in this study showed that when applied to a large patient cohort in which CTs were collected from multiple data sources, this radiomic feature method demonstrated poor performance, which may be due to the heterogeneity in image acquisition, reconstruction methods, or effects of post-processing.

Deep learning approaches have demonstrated impressive performance in recent years in medical fields such as automatic segmentation and diagnostic task such as lung nodule detection. Due to the fact that deep learning models are generally robust and can be applied to a wide variety of scenarios once properly trained with enough data, it has been previously applied to the task of survival prediction. In this project, we applied a ResNet-50 deep neural network, which took tumor segments (Tumor-CNN model) as input resulting in an AUC score of 0.6144. When analyzing the Tumor-CNN model’s performance from the medical perspective, we demonstrated that tumors contained the majority of prognostic information, yet adjacent non-tumor regions and their interactions with each other may have an effect on an individual patient’s survival. This hypothesis was based on our intuition that tumors spread from the primary sites *via* lymphatic drainage, hematogenous (*via* the vascular supply) or directly to the surrounding lungs (29). We therefore reasoned that such regional information can potentially be mapped *via* a graph representation method to represent the entire lung as input with an emphasis on the tumors as an additional node on an individual patient’s basis. Moreover, the best performance achieved by our GCN model demonstrated that using a relational data representation method can help improve the performance when compared to traditional deep learning models. To this end, our model demonstrated best accuracy in identifying high-risk patients, particularly on stage I patient group, demonstrating that features generated by GCN can find the survival-relevant information from early-stage patients’ CT image. The RFS Kaplan–Meier analysis revealed that the GCN approach also contained information that related to disease relapse, and combining that information from both of the above two aspects to analyze individual’s survival result likely contributes to improve performance.

On reviewing the whole process of our graph survival predictor formulation, all the steps were fully automated and could be easily applied to prospective patients in the future. Unlike radiomic approach, there was no need to specifically segment the tumors with our proposed method. By including regional information in graph structures likely contributes to improved prediction performance.

The results from our study have the following strengths. First, our dataset is large and has incorporated images from one large volume center with a standardized acquisition method, including contrast and non-contrast CT scans. Our model was found to be more generalizable as a result of training based on this large dataset with reasonable performance on external validation set. Second, our model’s whole procedure was fully automated. For example, segmenting the lung and airway took only a few seconds to obtain accurate results, which would allow ease of clinical translation. Finally, we conducted a series of experiments comparing our graph model to traditional model, widely used radiomic approaches and the most cutting-edge deep learning models, which supported our conclusion that the GCN models can outperform other conventional methods. We acknowledge, however, that due to differences in input features between these different models, comparison of performance may not be a fair one.

There are a few limitations in our study. First, while we achieved the best performance with the graph neural network, we did not investigate the model’s ability to discover new features, but it was apparent from our results that graph models have greater potential for future development due to their input of relational graph structures. Second, we used only CT images as input in this experiment because we have yet to develop a method for incorporating imaging data with demographic data such as age and gender information, which may improve the model’s performance. Some future work is being planned to improve the performance of our models. More anatomically relevant information could be incorporated into the graphs. For example, one could consider edge weight based on the location of the tumors for individual patients and create some other lung graph structures to better represent patients’ survival information. Furthermore, we intend to combine whole-slide imaging data from lung patients with CT data to better represent disease information in the future.

In this study, we presented a graph presentation model for describing CT data from early stage lung cancer patients and predicting their 5-year overall survival. Numerous experiments were conducted to compare our GCN model to traditional clinical model based on TNM staging, commonly used radiomic feature approaches, and state-of-the-art deep learning methods. We demonstrated that our graph methods performed significantly better compared with other existing models.

## Data Availability Statement

The original contributions presented in the study are included in the article/**Supplementary Materials**. Further inquiries can be directed to the corresponding authors.

## Ethics Statement

The studies involving human participants were reviewed and approved by Tongji University. The patients/participants provided their written informed consent to participate in this study.

## Author Contributions

Conception and design: JL, QD, and VV. Administrative support: QD and VV. Provision of study materials or patients: JL, FL, BF, and DL. Collection and assembly of data: JL and YL. Data analysis and interpretation: JL, YL, FH, and KSN. Manuscript writing: all authors. Final approval of manuscript: all authors. The corresponding author had full access to all the data in the study and had final responsibility for the decision to submit for publication.

## Conflict of Interest

The authors declare that the research was conducted in the absence of any commercial or financial relationships that could be construed as a potential conflict of interest.

## Publisher’s Note

All claims expressed in this article are solely those of the authors and do not necessarily represent those of their affiliated organizations, or those of the publisher, the editors and the reviewers. Any product that may be evaluated in this article, or claim that may be made by its manufacturer, is not guaranteed or endorsed by the publisher.
