# Supplementary material for: Imaging-Based Deep Graph Neural Networks for Survival Analysis in Early Stage Lung Cancer Using CT: A Multicenter Study
Source: Front Oncol. 2022 Jul 13;12:868186. doi: 10.3389/fonc.2022.868186 (PMC9351205; doi:10.3389/fonc.2022.868186)
Supplement: Supplementary file 1 [file DataSheet_1.docx]

Supplementary Material

# Scanner Parameters

Supplementary Material should be uploaded separately on submission. Please include any CT scans ranged from thoracic inlet to subcostal plane and were obtained before surgical resection from 2 CT machines: Brilliance (Philips Medical Systems Inc, Cleveland, OH) and SOMATOM Definition AS (Siemens Aktiengesell-schaft, Munich, Germany).

CT parameters of Brilliance (Philips Medical Systems Inc) were as follows: 64 × 1 mm acquisition; 0.75-second rotation time; slice width 1 mm; tube voltage, 120 kVp; tube current, 150 to 200 mA; lung window center: -700 Hounsfield units (HU), and window width:1200 HU; mediastinal window center: 60 HU and window width: 450 HU level; pitch: 0.906; and field of view (FOV): 350 mm.

CT parameters of the SOMATOM Definition AS (Siemens Aktiengesell-schaft) were as follows: 128 × 1 mm acquisition; 0.5-second rotation time; slice width: 1 mm; tube voltage: 120 kVp; tube current: 150 to 200 mA; lung window center: -700 HU and window width 1200 HU; and mediastinal window center: 60 HU and window width: 450 HU level; FOV: 300 mm; pitch: 1.2; and FOV: 350 mm. CT images were reconstructed into 0.67- to 1.25-mm section thicknesses according to a high-resolution algorithm.

# Follow-up Methods

Follow-up was conducted through outpatient examinations or telephone calls.

Chest CT scan and abdominal ultrasound/CT were performed on follow-up visits within a duration of 3, 6, and 12 months after operation and annually thereafter for 5 years. Magnetic resonance imaging for brain and bone scan were annually performed for 5 years or when the patient had signs or symptoms of recurrence.

# Four-point searching

# After we get the airway skeleton, we searched for 4 important points: root point, the center point, the left point, and the right point.

We first removed all disconnected componence in the skeleton.

Root point: a point has the minimal z position value in skeleton, and it only has neighbors that have larger z position values.

Center point: a point whose x, y and z position values are near the center of the whole image, and it has neighbors in three directions (up, left, and right).

Left point: a point whose x position value is smaller than center point, and it have at neighbors in two directions (up and down).

Right point: a point whose x position value is larger than center point, and it have at neighbors in two directions (up and down).

# Supplementary Tables 1: Performance of ML models, Tumor-CNN model and GCN models

| **Model** | **Accuracy  (95% CI)** | **Sensitivity  (95% CI)** | **Specificity  (95% CI)** | **Precision**  **(95% CI)** | **F_2_ Score**  **(95% CI)** |
| --- | --- | --- | --- | --- | --- |
| **DT-radiomics** | 0.699  (0.637 - 0.761) | 0.348  (0.227 - 0.986) | 0.796  (0.728 - 0.850) | 0.320  (0.207 - 0.458) | 0.341  (0.284 - 0.404) |
| **Tumor-CNN** | 0.704  (0.643 - 0.766) | 0.456  (0.321 – 0.598) | 0.772  (0.703 - 0.829) | 0.356  (0.246 - 0.483) | 0.432  (0.371 - 0.495) |
| **GCN** | 0.737  (0.678 - 0.796) | 0.697  (0.552 - 0.809) | 0.748  (0.678 - 0.808) | 0.432  (0.326 - 0.546) | 0.620  (0.560 - 0.677) |
